# A virus from *Aspergillus cibarius* with features of alpha- and betachrysoviruses

**DOI:** 10.1007/s11262-023-02043-6

**Published:** 2023-12-30

**Authors:** Tobias Lutz, Gitta Jutta Langer, Cornelia Heinze

**Affiliations:** 1 Institute of Plant Science and Microbiology, University of Hamburg, Molecular Phytopathology, Ohnhorststr. 18, 22609 Hamburg, Germany; 2 Nordwestdeutsche Forstliche Versuchsanstalt, Grätzelstr. 2, 37079 Göttingen, Germany

**Keywords:** Chrysovirus, Processing, *Aspergillus*, Mycovirus, dsRNA

## Abstract

From the ascomycete *Aspergillus cibarius* strain NW-FVA 2590, which was originally isolated from a root, associated with stem collar necrosis of *Fraxinus excelsior* L., a novel virus was isolated and characterized. Its genome is encoded on three monocistronic dsRNA segments ranging from 3683 bp (dsRNA 1) over 3093 (dsRNA 2) to 2902 bp (dsRNA 3), which are packed in isometric particles of around 35 nm. While the viral RdRp (P1) is encoded on segment 1, protein sequencing showed that two more structural proteins are present which are translated from dsRNA 2 (P2) and dsRNA 3 (P3) and possibly form the viral capsid. Additionally, P2 and P3 may undergo posttranslational modifications since the detected proteins bands deviated from the calculated sizes. Due to its phylogenetic position, the novel virus was grouped in the family of *Chrysoviridae* and was tentatively denominated as Aspergillus cibarius chrysovirus 1 (AcCV1). Due to its composition, biological properties and phylogenetic position, distant from the genera *Alphachrysovirus* and *Betachrysovirus*, we suggest to position AcCV1 in a proposed genus “*Gammachrysovirus*”*.*

**Supplementary Information:**

The online version contains supplementary material available at 10.1007/s11262-023-02043-6.

## Introduction

The genus *Aspergillus* P. Micheli ex Haller (*Aspergillaceae*, Eurotiales, Eurotiomycetidae, Eurotiomycetes, Pezizomycotina, Ascomycota, Fungi) consists of several hundred mold species found in various climates worldwide [1]. The genus comprises asexual spore-forming species, of which about one third are known to have a sexual Eurotium Link stage [2]. Especially in *Aspergilli*, many mycoviruses are consistently found since they are subject of investigations as ecological, economical and medical important fungi [3, 4]. In a review of Kotta-Loizou and Coutts [3] over 170 infected isolates of *Aspergillus* sp. from families us *Partitiviridae*, *Totiviridae* or *Chrysoviridae* were reported in 2017.

The family *Chrysoviridae* is grouped within the order of *Ghabrivirales* and accommodates the two genera *Alphachrysovirus* and *Betachrysovirus.* Their genome consists of three to seven monocistronic dsRNA elements, individually packed into isometric particles of around 40 nm, with an overall genomic size of 8.9 kbp – 16.0 kbp [5]. While betachrysoviruses harbor four to seven dsRNA segments and are mainly found in fungi as asco- and basidiomycetes, alphachrysoviruses are tri- or tetra-segmented and are additionally detected in plants and invertebrates [6–9]. According to the ICTV, the viral RdRp and a separate NTPase domain is encoded from ORF 1 (P1), and the main capsid from ORF 2 (P2). However, the capsid of cinquechrysoviruses in the *Betachrysovirus* genus can be composed of more structural proteins which can be processed at their C-termini, as it was shown for Fusarium graminearum virus-China 9 (FgV-ch9) [10] or for Magnaporthe oryzae chrysovirus 1 (MoCV1) [5, 11]. In most alphachrysoviruses, downstream of the conserved extreme 5’-terminus which often harbor an A-rich stretch, a highly preserved region of 45–70 nts is found and for some, ‘CAA’ repeats are present upstream of the translation initiation site [12]. These repeats are considered to function as translational enhancer similar to ‘CAA’ repeats in tobacco mosaic viruses (TMV) [5, 12, 13]. Some viruses from both chrysovirus genera have been reported to induce growth alterations or even hypovirulence to their hosts [14–18].

In our study, we provide the complete genome organization and sequence analysis of a novel tripartite dsRNA virus isolated from *A. cibarius*. Additionally, we give a deeper insight into the virus particle composition and its features. Based on sequence properties and phylogenetic position, the virus was tentatively grouped in the *Chrysoviridae* family and therefore it was named Aspergillus cibarius chrysovirus 1 (AcCV1). Due to its special properties which are found to be in between both genera and due to its distant relation to alpha- and betachrysoviruses, we propose to establish a new genus, “*Gammachrysovirus”*, to accommodate AcCV1.

## Material and methods

### Fungal isolates and cultivation

The *Aspergillus cibarius* strain NW-FVA 2590 was isolated from a necrotic root associated with stem collar necrosis of *Fraxinus excelsior* L. It was collected 2015 in the Bezirksförsterei Hils-Vogler-Ost in Stroit, corridor 3, parcel 100/9, in southern Lower Saxony, Germany, by M. Pfeffer. Isolation and identification was performed as described by Langer [19] by sequencing the internal transcribed spacer region (ITS; acc. ID: KU712229). Additionally, the strain was verified by sequencing the β-tubulin (BenA; acc. ID: OR612018) and the calmodulin (CaM; acc. ID: OR612016 and OR612017) regions [20–23]. Mycelium was cultivated at 28 °C on Czapek Yeast Autolysate agar supplemented with 20% w/v sucrose (CYA20) according to HiMedia Laboratories (Mumbai, Maharashtra, India) and Siqueira [24]. The phenotype of the fungus corresponded to the one described by Hong [25].

### Isolation of virus like particles, protein analysis and electron microscopy

Virus-like particles (VLPs) were enriched according to Aoki et al. [26] as described in Lutz et al. [10] with modifications. Briefly, 1 to 3 g mycelium was crushed under liquid nitrogen. The powder was resuspended in 100 ml 0.1 M sodium phosphate (pH 7) and coarse material was removed by centrifugation (8000×*g*, 20 min). The supernatant was clarified once with 20% (v/v) chloroform/n-butanol (1:2). The upper phase was stirred in the presence of 8% (w/v) polyethylene glycol (PEG6000) and 1% (w/v) NaCl overnight at 4 °C. The precipitate was sedimented (10,000×*g*, 20 min) and resuspended in 0.05 M sodium phosphate (pH 7), which was layered on top of 20% (w/v) sucrose in 0.05 M sodium phosphate (pH 7) and centrifuged (105,000×*g*, 2 h). The pellet was resuspended in in 0.05 M sodium phosphate (pH 7) and stored at − 70 °C.

Proteins of purified particles were separated by SDS-PAGE according to Laemmli [27] and stained with Coomassie Brilliant Blue [28]. Bands were cut from the gel and peptides were sequenced with LC–MS/MS by a nano-liquid chromatography system (Dionex UltiMate™ 3000 RSLCnano, ThermoFisher Scientific, Waltham, Massachusetts, USA) and analyzed by means of the Proteome Discoverer 2.0 (ThermoFisher Scientific) by the Universitätsklinikum Hamburg-Eppendorf (UKE, Hamburg, Germany).

Additionally, VLPs were examined by transmission electron microscopy (LEO 906E, Zeiss, Germany) with 2% (w/v) uranyl acetate contrasting.

### Isolation of dsRNA and sequence analysis

Nucleic acids were extracted from particles using the Double-RNA—Viral dsRNA Extraction Kit (iNtRON Biotechnology, Seongnam-Si, South Korea). Isolated dsRNA was examined by 1% (w/v) agarose gel electrophoresis and subjected to next-generation sequencing. Libraries were prepared using a Nextera XT DNA Library Preparation Kit (Illumina Inc., San Diego, CA, USA) and sequenced on a NextSeq 2000 (Illumina Inc., San Diego, CA, USA) instrument at the Leibniz Institute DSMZ (Braunschweig, Germany) as paired-end reads (2 × 151). De novo assembly was performed and contigs were analyzed using Geneious Prime software version 2021.2.2 (RRID:SCR_010519). The 5′- and 3′-termini of each segment were determined by the single-primer amplification technique (SPAT) as it was described by Zhong [29], using an oligonucleotide with a phosphorylated 5′-terminus and a 2′,3′-dideoxyC group (23ddC) at the 3′-terminus as a blocker to prevent self-ligation (5′-PO4-TCTCTTCGTGGGCTCTTGCG-23ddC-3′). Sequence specific primers are displayed in Supplementary Table 1. Amplicons were cloned into pGEM®-T Vector (Promega Corporation, Madison, Wisconsin, USA) and were sequenced.

### Sequence and phylogenetic analysis

Nucleic acid sequences and ORFs were analyzed using SnapGene version 6.2 (RRID:SCR_015052) and BLAST on the NCBI website [30]. Sequence alignments and phylogenetic analysis were performed using MEGA X version 10.2.4 (RRID:SCR_023471) [31] using the Clustal Omega [32–34] and the MUSCLE [35, 36] algorithm in default settings. Conserved protein domains were identified by conserved domain database (CDD) search on the NCBI website [37–40].

The evolutionary history was inferred by using the Maximum Likelihood method and the Le and Gascuel model [41]. The tree with the highest log likelihood (− 43,498.30) is shown. The percentage of trees in which the associated taxa clustered together is shown next to the branches. Initial trees for the heuristic search were obtained automatically by applying Neighbor-Join and BioNJ algorithms to a matrix of pairwise distances estimated using the JTT model, and then selecting the topology with superior log likelihood value. A discrete Gamma distribution was used to model evolutionary rate differences among sites (5 categories; + G, parameter = 2.3244). The rate variation model allowed for some sites to be evolutionarily invariable ([+ I], 2.56% sites). The tree is drawn to scale, with branch lengths measured in the number of substitutions per site. This analysis involved 30 amino acid sequences. There were a total of 1248 positions in the final dataset.

Figures were generated and edited using Inkscape version 1.2.2 (RRID:SCR_014479), UGENE version 1.32.0 (RRID:SCR_005579) [42] and SnapGene.

## Results

### Sequence properties

**Fig. 1:**
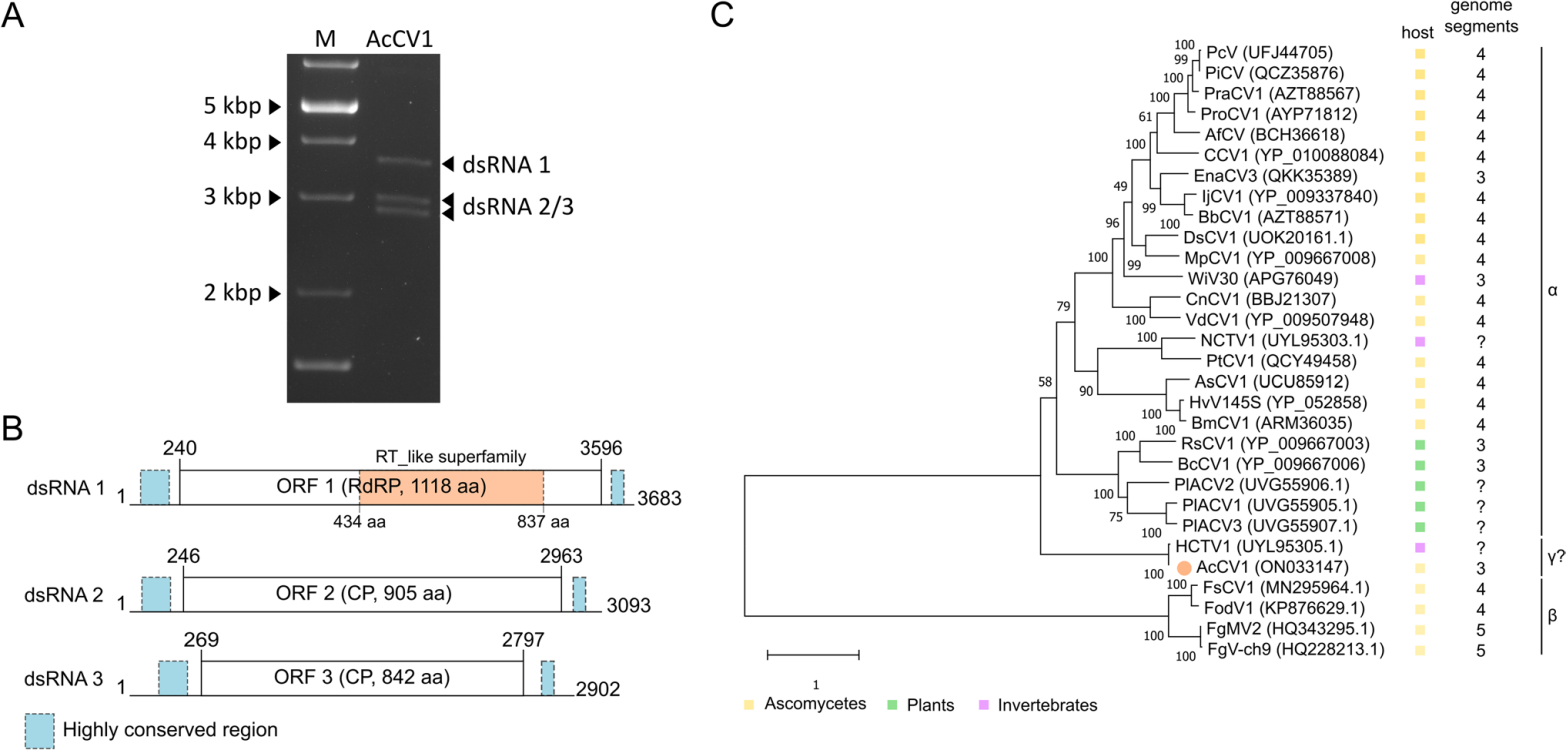
Examination of dsRNA by agarose gel electrophoresis 1% (w/v), the coding strategy and phylogenetic analysis of AcCV1. **A** Extracted dsRNA of AcCV1. M, GeneRuler 1 kb Plus DNA Ladder (ThermoFisher Scientific). The sizes of the marker are indicated on the left. **B** The dsRNA segments are displayed as horizontal lines with their respective UTRs at each terminus. ORFs are represented as boxes with start and stop codon positions indicated above the boxes. The conserved RT_like superfamily is indicated with an orange box within ORF 1 and highly conserved regions are annotated as light blue boxes above the UTRs. Note that the genome organization is not drawn to scale. **C** Maximum Likelihood analysis of AcCV1 with 1000 bootstrap replicates whose values are displayed at the nodes. The bar corresponds to the genetic distance. The different hosts of the viruses are indicated with boxes: yellow (ascomycetes), green (plants) and pink (invertebrates). Additionally, the number of segments and their affiliation to either alpha(α)- or beta(β)chrysoviruses is indicated on the right. The names of viruses are abbreviated as follows: AfCV: Aspergillus fumigatusc Chrysovirus; AcCV1: Aspergillus cibarius chrysovirus 1; AsCV1: Alternaria solani chrysovirus 1; BbCV1: Beauveria bassiana chrysovirus 1; BcCV1: Brassica campestris chrysovirus 1; BmCV1: Bipolaris maydis chrysovirus 1; CCV1: Chrysothrix chrysovirus 1; CnCV1: Cryphonectria nitschkei chrysovirus 1; DsCV1: Diplodia seriata chrysovirus 1; DsCV1: Diplodia seriata chrysovirus 1; EnaCV3: Erysiphe necator associated chrysovirus 3; FgMV2: Fusarium graminearum dsRNA mycovirus-2; FgV-ch9: Fusarium graminearum virus-China 9; FodV1: Fusarium oxysporum f. sp. dianthi mycovirus 1; FsCV1: Fusarium sacchari chrysovirus 1 strain FZ04; HCTV1: Hulunbuir Chrys tick virus 1; HvV145S: Helminthosporium victoriae 145S virus; IjCV1: Isaria javanica chrysovirus 1; MpCV1: Macrophomina phaseolina chrysovirus 1; NCTV1: Nanning Chrys tick virus 1; PcV: Penicillium chrysogenum virus; PiCV: Penicillium italicum Chrysovirus; PlACV1-3: Poaceae Liege alphachrysovirus 1–3; PraCV1: Penicillium raistrickii chrysovirus 1; ProCV1: Penicillium roseopurpureum chrysovirus 1; PtCV1: Pestalotiopsis theae chrysovirus 1; RsCV1: Raphanus sativas chrysovirus 1; VdCV1: Verticillium dahliae chrysovirus 1; WIV30: Wuhan insect virus 30

The complete genome sequence of AcCV 1 is composed of three monocistronic dsRNA segments (Fig. 1B) and has been deposited in the GenBank database (accession ID: ON033147–ON033149). In total, the genome of AcCV1 consists of 9678 bp. The GC content of each segment ranges from 45% (dsRNA 1) to 47 (dsRNA 2 and dsRNA 3). The sequenced segments corresponded in number and size to the bands detected by agarose gel electrophoresis, which showed bands ranging from 3.7 to 2.9 kbp (Fig. 1A).

Segment 1 is 3683 bp in length. Its ORF spans from nucleotide position 240 to 2596 and encodes a protein with 1119 aa and a calculated molecular weight of 126.66 kDa. A BLASTp search showed the highest degree of similarity to the putative RdRp of the Hulunbuir Chrys tick virus 1 (HCTV1; 99.11% aa sequence identity, E-value 0.0, acc. ID: UYL95305.1) and of the Penicillium roseopurpureum chrysovirus 1 (PrCV1; 38.73% aa sequence identity, E-value 0.0, acc. ID: AYP71812.1). A conserved RdRp motif of the RT_like superfamily was detected by CDD search between aa _434_H-W_837_ (acc. ID: cl02808 with an E-value of 3.69e-39) and the conserved RdRp motif VI was located at aa _771_GDD_773_.

Segment 2 is 3093 bp in length. Its ORF spans from nucleotide position 246 to 2963 and encodes a protein with 906 aa and a calculated molecular weight of 100.78 kDa. A BLASTp search showed the highest degree of similarity (30.13% aa sequence identity, E-value 7e-46) to the capsid protein of the Alphachrysovirus aspergilli (ACVA; acc. ID: YP_009508105.1).

Segment 3 is 2902 bp in length. Its ORF spans from nucleotide position 269 to 2797 and encodes a protein with 843 aa and a calculated molecular weight of 94.02 kDa. A BLASTp search showed the highest degree of similarity (27.42% aa sequence identity, E-value 3e-67) to P4 of the Macrophomina phaseolina chrysovirus 1 (MpChrV1; accession ID: YP_009667011.1).

The 5’ UTRs of each segment range in length between 239 (dsRNA 1), 245 bp (dsRNA 2) and 268 bp (dsRNA 3). All segments possess an identical extreme 5’-terminus (5′-TGAAAAAATTACA-3′) (Supplementary Fig. S 1) and highly conserved stretches at the 5′- (49 identical nts) and 3′-termini (29 identical nts) (Fig. 1B, light blue; Supplementary Fig. S 1). The 3′-termini of each segment range from 87 bp (dsRNA 1), 130 bp (dsRNA 2) and 105 bp (dsRNA 3) and the extreme ends are conserved (5′-AAGCGC-3′) (Supplementary Fig. S 1). The ‘CAA’ repeats were only poorly detected in the 5′-UTR segment 2 and not at all in 5′-UTRs of segments 1 and 3.

To determine the taxonomic position of AcCV1, a Maximum Likelihood tree was constructed based on a BLASTp search of the sequence of the putative RdRp (P1) of AcCV1. As an outgroup, the RdRp sequences of the betachrysoviruses Botrytis cinerea RNA virus 2, Botrytis cinerea mycovirus 4, Fusarium graminearum mycovirus 2 and Fusarium graminearum virus-China 9 were included. The novel AcCV1 clusters together with HCTV1 and builds an own clade between alphachrysoviruses isolated from plants and betachrysoviruses from *Fusarium* sp.

### Protein properties

**Fig. 2:**
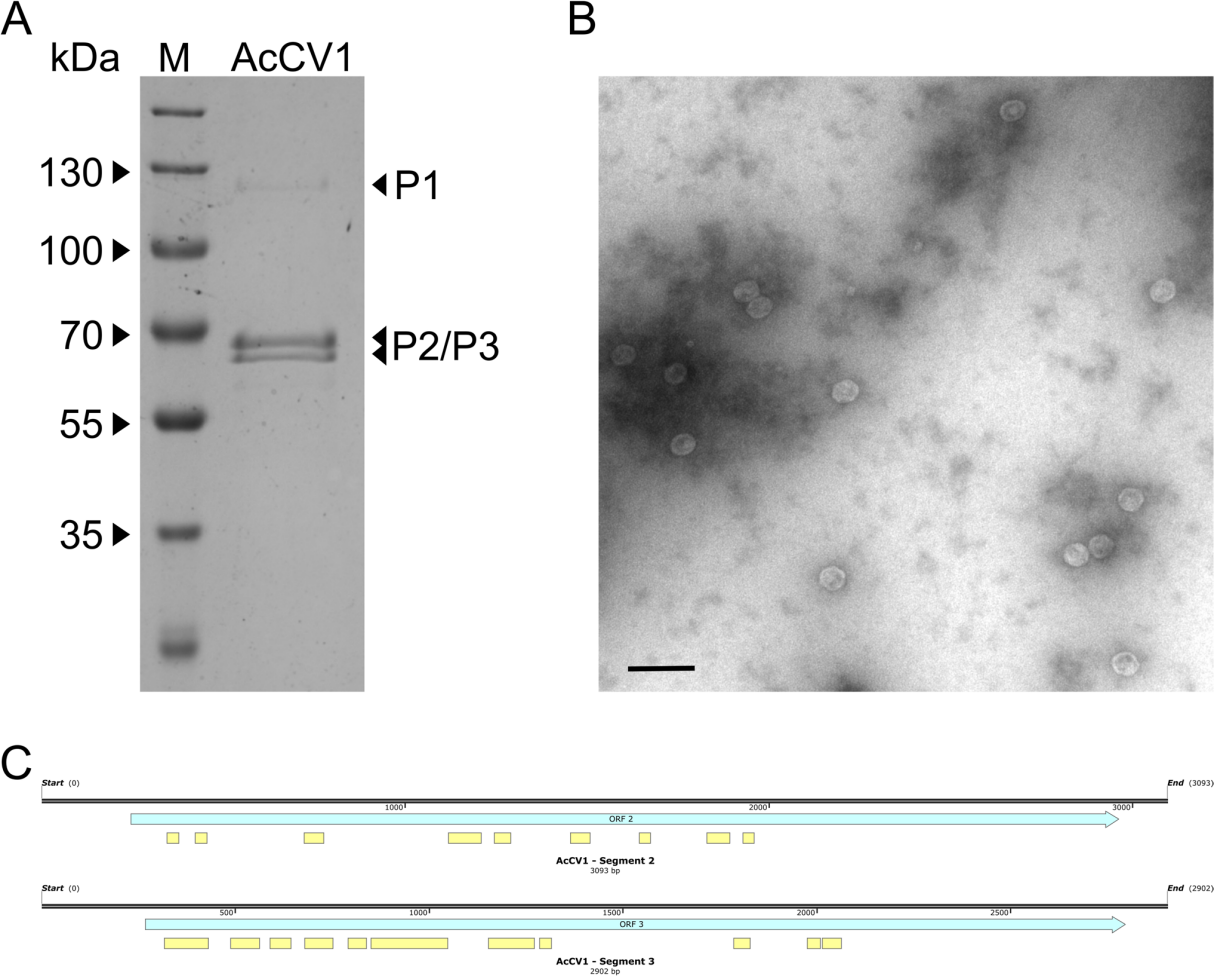
Examination of VLPs by SDS-PAGE (12.5% w/v) and Coomassie Brilliant Blue staining, transmission electron micrograph of VLPs contrasted with 2% (w/v) uranyl acetate and the peptides mapped against the aa sequences of P2 and P3 after protein sequencing by nano-liquid chromatography. **A** Protein pattern of VLPs of AcCV1. M, PageRuler Prestained Protein Ladder (ThermoFisher Scientific). The sizes of the marker are indicated on the left. **B** Ultra-structures obtained from VLPs of AcCV1. The black bar corresponds to 100 nm. **C** Against the aa sequence of P2 (upper) and P3 (lower) mapped peptides after protein sequencing. The ORF of each segment is highlighted in light blue and the peptides are annotated underneath the ORF in light yellow

To further study the viral composition and structure of AcCV1, VLPs were purified and examined by SDS-PAGE and Coomassie Brilliant Blue staining as well as transmission electron microscopy. Isometric particles with an average size of around 35-nm were observed (Fig. 2B). The protein pattern of the extracted VLPs is shown in Fig. 2A. One distinct band which corresponds to the RdRp (P1) at around 125 kDa was visible and additionally, two bands at around 63 kDa (P63) and 58 kDa (P58) were detected. Since the proteins of P2 and P3 were calculated between 100 and 95 kDa, these bands were cut out and sequenced by nano-liquid chromatography. Several N-terminal peptides were mapped against the aa sequences of P2 and P3 (Fig. 2C) and P63 was assigned to P2 and P58 to P3.

## Discussion

Species of the genus *Aspergillus* belong to the best studied fungi worldwide and over 170 virus infections already have been described until 2017 [3, 4], e.g. in the species *A. niger, A. flavus*, and *A. fumigatus*. This is the first report of a mycoviral-infection found in *A. cibarius*. Due to its genomic properties, particle composition and phylogenetic position, we grouped it in the *Chrysoviridae* family and tentatively denominated it as Aspergillus cibarius chrysovirus 1 (AcCV1). However, no distinct assignment of either of the two genera, *Alphachrysovirus* and *Betachrysovirus*, was possible.

The genome of AcCV1 is tri-segmented and comprises 9678 bp with one single ORF on each segment flanked by non-coding regions. The extreme termini of the segments are conserved and additionally the extreme 5′-termini have A-rich stretches which is typical for many viruses of both chrysovirus genera. The ‘CAA’ repeats, which function as a translation enhancer in several RNA viruses as TMV [13] were not only found in quadriviruses [43, 44] and partitiviruses [45], but were also reported for both chrysovirus genera like the PcV [12], the Amasya cherry disease associated chrysovirus (ACDACV) [46] in the genus *Alphachrysovirus* and the Botryosphaeria dothidea chrysovirus 1 (BdCV1) [47] in the *Betachrysovirus* genus. In AcCV1, these ‘CAA’ repeats were only poorly present in segment 2 and are missing in the segments 1 and 3, which is consistent with the findings of Ding et al. [48] for BdCV1-G1. Therefore, this feature does not seem like a characteristic for the family of *Chrysoviridae,* but rather of RNA viruses which may have acquired this trait independent of their respective families.

Some chrysoviruses encode conserved motifs on their segments. For instance, the motifs which form the conserved core of the ovarian tumor gene-like superfamily of predicted cysteine proteases (OTU) are present in most alphachrysovirus-P4 [5, 46]. Even though the tri-segmented chrysovirus Raphanus sativus chrysovirus 1 (RsCV1) isolated from *Raphanus sativus* harbors on its P3 the conserved motif PGDG(K/S)CGXHA as one of four motifs of the OTU superfamily [8], it is not present in the tri-segmented and closely related Brassica campestris chrysovirus 1 (BcCV1) [9]. Although this motif was not found in AcCV1, we cannot exclude that P2 or P3 might have a comparable function. Similar to the ‘CAA’ repeats in the viral genome, motifs of OTU superfamily seem rather dispersed across the different species within the *Alphachrysovirus* genus and are absent in betachrysoviruses. Both of these features, the OTU and the ‘CAA’ repeats, may have been acquired by horizontal gene transfer (HGT) as it was discussed for different motifs in different mycoviruses e.g. a Phytoreo_S7 domain in non-phytoreoviruses by Liu et al. [49] or a papain-like protease domain on dsRNA 2 of Sclerotinia sclerotiorum megabirnavirus 1 (SsMBV1) [50]. Both motifs are absent in AcCV1 or only poorly present.

Interestingly, AcCV1 clusters in a distinct clade with Hulunbuir Chrys tick virus 1 (HCTV1) which sequence was determined by the analysis of metagenomics and was exclusively found by BLASTp search. Sadly, no further information about this virus is available, especially it would be of interest whether the HCTV1 is a genuine insect virus or rather a mycovirus which host was present in the tick. The group which clusters next to AcCV1 and HCTV1 is formed by five alphachrysoviruses sequenced from plants. However, only for the genome of RasCV1 a detailed description is available and absence of any fungal infection was confirmed [8]. The genomes of Poaceae Liege alphachrysoviruses 1-3 (PlACV1-3), which were also present in the phylogenetic group next to AcCV1, were mapped in an analysis of the virome of different *Poaceae* communities in highly-, medium- and non-managed areas by metagenomics [7]. The authors found a higher viral prevalence in less-managed plant communities and the PlACV1-3 were exclusively detected in fungicide-free pastures and grasslands. Therefore, one can speculate that PIACV1-3 are genuine mycoviruses and only the tri-segmented RasCV1 is a confirmed plant virus. Since only the sequences of the viral RdRps of PlACV1-3 are deposited, it is tough to draw further conclusions regarding genome segments or particle composition. Overall, AcCV1 clusters within the genus *Alphachrysovirus* but is only distantly related.

Virus like particles were isolated and an isometric structure with size of around 35 nm was observed which corresponds to other chrysoviruses [5]. After examination of the protein pattern of VLPs three distinct bands were observed. To further investigate the particle composition of AcCV1, the protein pattern was analyzed and revealed three distinct bands. Sequencing of these addressed the band of 125 kDa to P1 with the expected size and the two lower bands to P2 and P3, respectively. It was unexpected that all three proteins encoded by AcCV1 are structural. The capsid of alphachrysoviruses and of betachrysoviruses are usually built of subunits of proteins encoded on one ORF. Only the genome of cinquechrysoviruses often codes for two capsid proteins, like it was experimentally shown e.g. for MoCV1 and FgV-ch9 [5, 10, 11].

In only few reports of chrysoviruses, data of protein patterns of VLPs were presented. In some of these reports, the size of the bands were in accordance with the MW derived from the respective ORF [12, 51, 52]. In other publications [8, 10, 11, 17, 18, 29, 47, 48, 53], the protein patterns of VLPs of both genera differed from the calculated sizes. For example, Li et al. [8] speculated for the main capsid protein P2 of the plant-alphachrysovirus RasCV1 that it undergoes auto-proteolytical modification. Similarly, Shahi et al. [53] discussed the viral structural proteins to be degraded during preparation of VLPs. The protein composition of MoCV1 was studied in more detail by Urayama et al. [11]. The authors were able to show by the use of Edman degradation and the use of antisera that the virus particle is built of P3 and P4, while P3 was lacking the C-terminus and P4 its N- and C-terminal domain. They discussed that both proteins undergo degradation by posttranslational cleavage or posttranslational modifications which might occur rapidly during purification or intracellularly. Later, Lutz et al. [10] proved that the structural proteins P2 and P3 of FgV-ch9 and P3 of FodCV1 are C-terminally processed by host factors in a directed manner. They also showed that P3 is processed by factors being present in eukaryotes such as plant sap, which indicates that the VLPs of RasCV1 might also be processed by a host encoded factor.

**Fig. 3:**
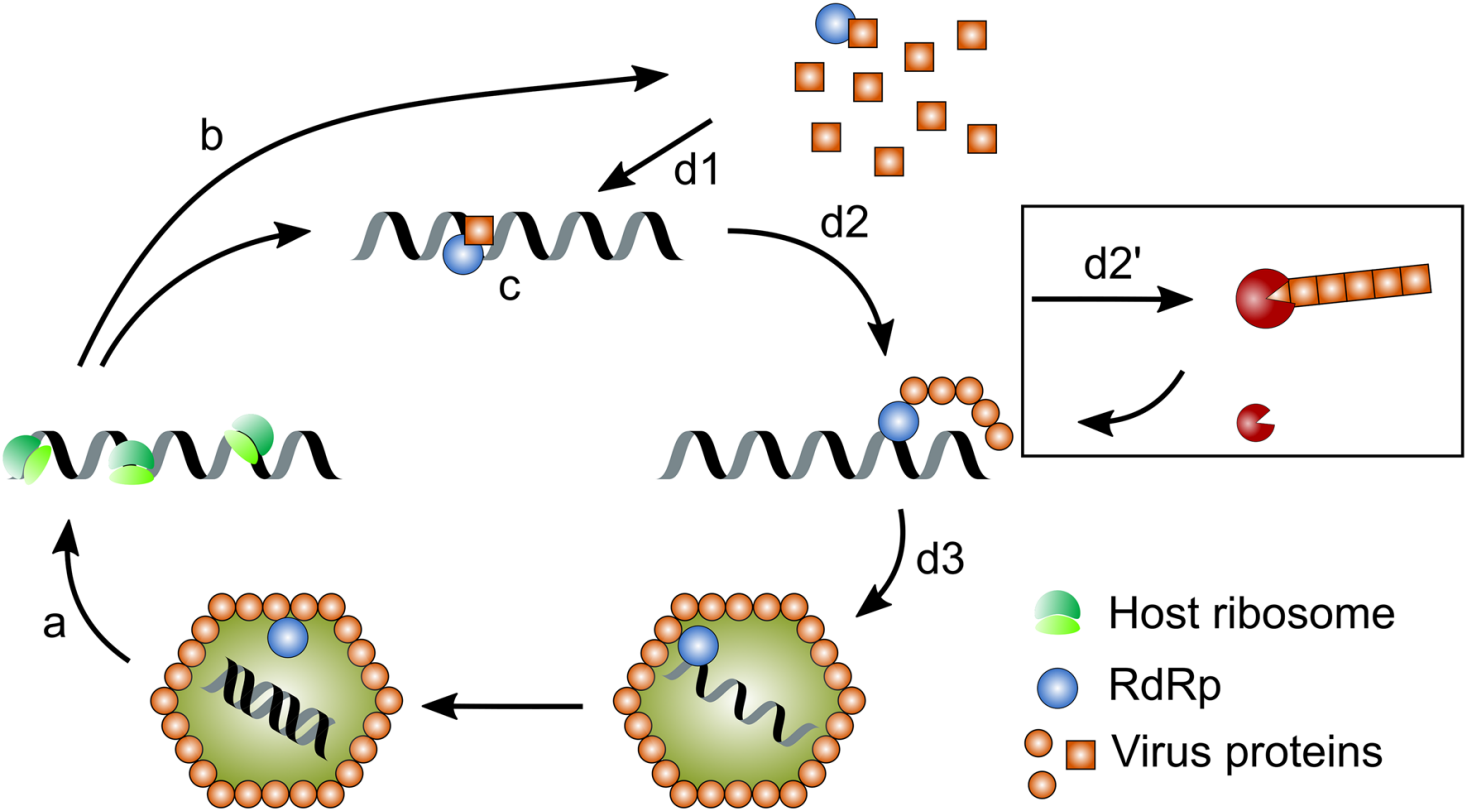
Schematic illustration of the replication cycle of FgV-ch9 and AcCV1, which was modified from Schmitt and Breinig [54]. **a**: The (+)ssRNA gets released in to the cytoplasm of the host. **b**: Host ribosomes attach to the ready to use RNA and start the translation. **c** and **d1**: The newly synthesized RdRp binds to the (+)ssRNA. **d2** and **d2’**: The putative capsid proteins are processed by unknown host factors and attach to the RdRP-(+)ssRNA complex. **d3**: The final virus particle is built and the RdRp synthesizes the complementary strand within the particle

Since the two structural proteins P2 and P3 of AcCV1 deviate from their calculated sizes, we speculate that the they undergo posttranslational processing to form the final capsid as it was shown by Lutz et al. [10] and propose that the replication cycle of dsRNA viruses which was postulated by Schmitt and Breinig [54], can be complemented for chrysoviruses with processed capsids by a distinct processing stage (Fig. 3).

In conclusion, we reported a tri-segmented alphachrysovirus which harbors two putative processed capsid proteins and opens a new phylogenetic clade together with the HCTV1. Since biological properties of both genera are present and due to the distant relation to other members of the alpha- or betacchrysovirus genus, we suggest the establishment of the genus “*Gammachrysovirus”* to harbor AcCV1 and HCTV1.

### Supplementary Information

Below is the link to the electronic supplementary material.

Supplementary file1 (DOCX 123 KB)

## Data Availability

Not applicable.
