# Supplementary material for: A virus from Aspergillus cibarius with features of alpha- and betachrysoviruses
Source: Virus Genes. 2023 Dec 30;60(1):71–9. doi: 10.1007/s11262-023-02043-6 (PMC10861612; doi:10.1007/s11262-023-02043-6)
Supplement: Supplementary file 1 — Supplementary file1 (DOCX 123 KB) [file 11262_2023_2043_MOESM1_ESM.docx]

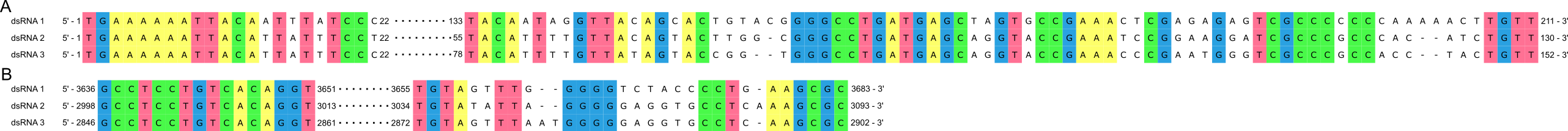


Supplementary Figure S 1: Alignment of the 5’- and 3’ NTRs of dsRNA 1-3 of AcCV1.

Supplementary Table 1: Oligonucleotides used in this study.

| Denomination | Sequence 5‘->3‘ |
| --- | --- |
| 2590-RNA1-I-REV | GCTTCAGCATACCCTAGCC |
| 2590-RNA1-II-FW | AAATGGGCTAATGGGCCG |
| 2590-RNA2-I-REV | TTCACGGCAGCCAGATGAGC |
| 2590-RNA2-II-FW | AGCCAAGGAAGGGTAGGACAGG |
| 2590-RNA3-I-FW | TTCATGGTTTGCAAGGCGCG |
| 2590-RNA3-II-FW | TTAGCTGGACTTTGCACGGCC |
